# Urinary proteomics links keratan sulfate degradation and lysosomal enzymes to early type 1 diabetes

**DOI:** 10.1371/journal.pone.0233639

**Published:** 2020-05-26

**Authors:** Julie A. D. Van, Sergi Clotet-Freixas, Anne-Christin Hauschild, Ihor Batruch, Igor Jurisica, Yesmino Elia, Farid H. Mahmud, Etienne Sochett, Eleftherios P. Diamandis, James W. Scholey, Ana Konvalinka

**Affiliations:** 1 Institute of Medical Science, University of Toronto, Toronto, Canada; 2 Toronto General Hospital Research Institute, University Health Network, Toronto, Canada; 3 Krembil Research Institute, University Health Network, Toronto, Canada; 4 Department of Mathematics & Computer Science, University of Marburg, Marburg, Germany; 5 Department of Laboratory Medicine and Pathobiology, Lunenfeld-Tanenbaum Research Institute, Mount Sinai Hospital, University of Toronto, Toronto, Canada; 6 Department of Computer Science, University of Toronto, Toronto, Canada; 7 Institute of Neuroimmunology, Slovak Academy of Sciences, Bratislava, Slovakia; 8 Hospital for Sick Children, Toronto, Ontario, Canada; 9 Department of Clinical Biochemistry, University Health Network, University of Toronto, Toronto, Canada; 10 Division of Nephrology, Department of Medicine, University Health Network, Toronto, Ontario, Canada; International University of Health and Welfare, School of Medicine, JAPAN

## Abstract

Diabetes is the leading cause of end-stage renal disease worldwide. Our understanding of the early kidney response to chronic hyperglycemia remains incomplete. To address this, we first investigated the urinary proteomes of otherwise healthy youths with and without type 1 diabetes and subsequently examined the enriched pathways that might be dysregulated in early disease using systems biology approaches. This cross-sectional study included two separate cohorts for the discovery (*N* = 30) and internal validation (*N* = 30) of differentially excreted proteins. Discovery proteomics was performed on a Q Exactive Plus hybrid quadrupole-orbitrap mass spectrometer. We then searched the pathDIP, KEGG, and Reactome databases to identify enriched pathways in early diabetes; the Integrated Interactions Database to retrieve protein-protein interaction data; and the PubMed database to compare fold changes of our signature proteins with those published in similarly designed studies. Proteins were selected for internal validation based on pathway enrichment and availability of commercial enzyme-linked immunosorbent assay kits. Of the 2451 proteins identified, 576 were quantified in all samples from the discovery cohort; 34 comprised the urinary signature for early diabetes after Benjamini-Hochberg adjustment (*Q* < 0.05). The top pathways associated with this signature included lysosome, glycosaminoglycan degradation, and innate immune system (*Q* < 0.01). Notably, all enzymes involved in keratan sulfate degradation were significantly elevated in urines from youths with diabetes (|fold change| > 1.6). Increased urinary excretion of monocyte differentiation antigen CD14, hexosaminidase A, and lumican was also observed in the validation cohort (*P* < 0.05). Twenty-one proteins from our signature have been reported elsewhere as potential mediators of early diabetes. In this study, we identified a urinary proteomic signature for early type 1 diabetes, of which lysosomal enzymes were major constituents. Our findings highlight novel pathways such as keratan sulfate degradation in the early kidney response to hyperglycemia.

## Introduction

Diabetes is the leading cause of end-stage renal disease worldwide. In the clinic, an early sign of diabetic kidney injury is microalbuminuria, which has traditionally been regarded as a defining point in the course of disease [1, 2]. Recent evidence however has demonstrated that kidney function may deteriorate in the absence of proteinuria [3, 4] and that microalbuminuria may revert back to normal urinary albumin excretion rates over time [5–7]. These findings undermine the clinical utility of microalbuminuria as a reliable predictor of disease. Therefore, important changes occur in the diabetic kidney long before clinical manifestations of injury.

Our modern understanding of early diabetic kidney injury was initially constructed using histopathological examinations from the 1980s [8, 9]. Notable features of the diabetic kidney under the microscope include mesangial expansion, glomerular basement membrane thickening, and podocyte loss, which are often present long before the onset of microalbuminuria or decline in kidney function [10]. Building on this framework has been difficult, as kidney biopsies carry significant morbidity and are typically performed in cases of advanced or atypical diabetic kidney disease. Accordingly, major gaps in knowledge continue to exist and present as barriers to improving care and delivering targeted treatments.

Urinary proteomics could offer novels insights into the pathogenesis of early diabetic kidney injury for three key reasons [11]. First, urine represents a suitable and non-invasive alternative to biopsies because it is directly produced by the kidneys. At this early stage of injury, the permselective barrier of the kidney remains largely intact, preventing the filtration of large macromolecules (>20 kDa) into urine. Proteins detected in urine are thus more likely to originate from the kidneys, ureter, and bladder than from the circulation. Second, this approach enables the broad characterization of thousands of proteins from relatively low volumes of urine [12]. Advances in mass spectrometry techniques have also significantly improved in accuracy and sensitivity in recent years. Third, proteomic analyses produce large datasets that can be further dissected *in silico* to extract information on tissue origins, protein-protein networks, and involvement in pathways and reactions. Therefore, to examine the early renal response to chronic hyperglycemia, we first conducted a proteomic investigation into the urines from otherwise healthy youths with and without type 1 diabetes and subsequently applied systems biology approaches to examine the early, dysregulated pathways that are overrepresented by the differentially excreted proteins.

## Materials and methods

### Study design and population

Our examination of the early kidney response to chronic hyperglycemia relies on the careful selection of an appropriate study population. Complete details of the study criteria have been described elsewhere in a peptidomic analysis [13]. Briefly, all participants were 19 years of age or younger; free of significant comorbidity including hypertension and proteinuria; and not using corticosteroid, anti-hypertensive, or anti-inflammatory medications. Youths with type 1 diabetes were considered to be in the earliest and uncomplicated stage of the natural history of diabetic kidney disease. They were recruited and initially screen from multiple diabetes clinics in the Greater Toronto Area; while youths without diabetes were either family members of those with type 1 diabetes or healthy volunteers recruited at the Hospital for Sick Children and Toronto General Hospital.

This cross-sectional study includes a discovery cohort (*N* = 30) and an internal validation cohort (*N* = 30). The primary exposure variable is diabetes status. Each participant provided a single urine sample for the study. To determine the appropriate cohort size, we performed power calculations using the following parameters in G*Power software: study power of 80%, the independent two-sample *t*-test and Benjamini-Hochberg (BH) correction with a false detection rate (FDR) of 0.0001, and an effect size *d* of 2. In each cohort, thirty samples were collected from 15 otherwise healthy youths with type 1 diabetes and 15 non-diabetic peers. Thus, a total of 60 second-morning midstream urine samples was collected from 60 youths.

Clinical characteristics of both cohorts at time of urine collection are summarized in Table 1. Groups in the discovery cohort were matched according to age (± 1 year) and sex; groups in the validation cohort were matched according to age (± 1 year) only. The research ethics boards at the Hospital for Sick Children and Mount Sinai Hospital approved this study. In accordance with the Declaration of Helsinki, written informed consent was obtained from the legal guardians, next-of-kin, or caretakers of youths under the age of 16 years, while said youths provided assent. Youths aged 16 and older with capacity to understand the study information provided complete written and informed consent to participate in the study.

**Table 1:** Clinical characteristics of both cohorts at time of urine collection.

	Discovery Cohort		Validation Cohort	
Clinical Characteristics	Youths without T1D (*N* = 15)	Youths with T1D (*N* = 15)	Youths without T1D (*N* = 15)	Youths with T1D (*N* = 15)
**Age (years)**	16.0 ± 1.8	15.7 ± 1.8	16.0 ± 1.2	16.5 ± 1.1
**Sex (females/males)**	6 / 9	6 / 9	5 / 10	6 / 9
**HbA1c (%)**	n.m.	8.9 ± 1.5	5.1 ± 0.3	9.1 ±1.6
**Diabetes duration (years)**	n.a.	9.7 ± 2.9	n.a.	10.4 ± 2.9
**ACR (mg/mmol)**	n.m.	0.8 ± 0.5	0.6 ± 0.2	1.1 ± 1.1
**eGFR (ml/min/1.73m**^**2**^**)**	n.m.	118 ± 18	102 ± 15	115 ± 24

Data is presented as mean ± standard deviation, except for sex (frequency). *P* values are shown between youths with and without type 1 diabetes (T1D). HbA1c, glycated hemoglobin; ACR, albumin/creatinine ratio; eGFR, estimated glomerular filtration rate; n.m., not measured; n.a., not applicable.

### Collection, handling, and storage of urines

Second-morning, midstream urines were collected, handled, and stored in accordance with the Standard Protocol for Urine Collection and Storage created by the Human Kidney and Urine Proteome Project (HKUPP) and the Human Proteome Organization (HUPO) [14]. Following collection, all fresh urine samples were kept at 4°C until further processing. All urine samples were centrifuged at 1000 g for 10 minutes to remove intact cells and debris. This initial processing step was completed within 3 hours of urine collection to obviate the need for urine preservatives. Samples were de-identified and randomized so that investigators were blinded to experimental groups during processing.

### Discovery proteomics

**Fig 1:**
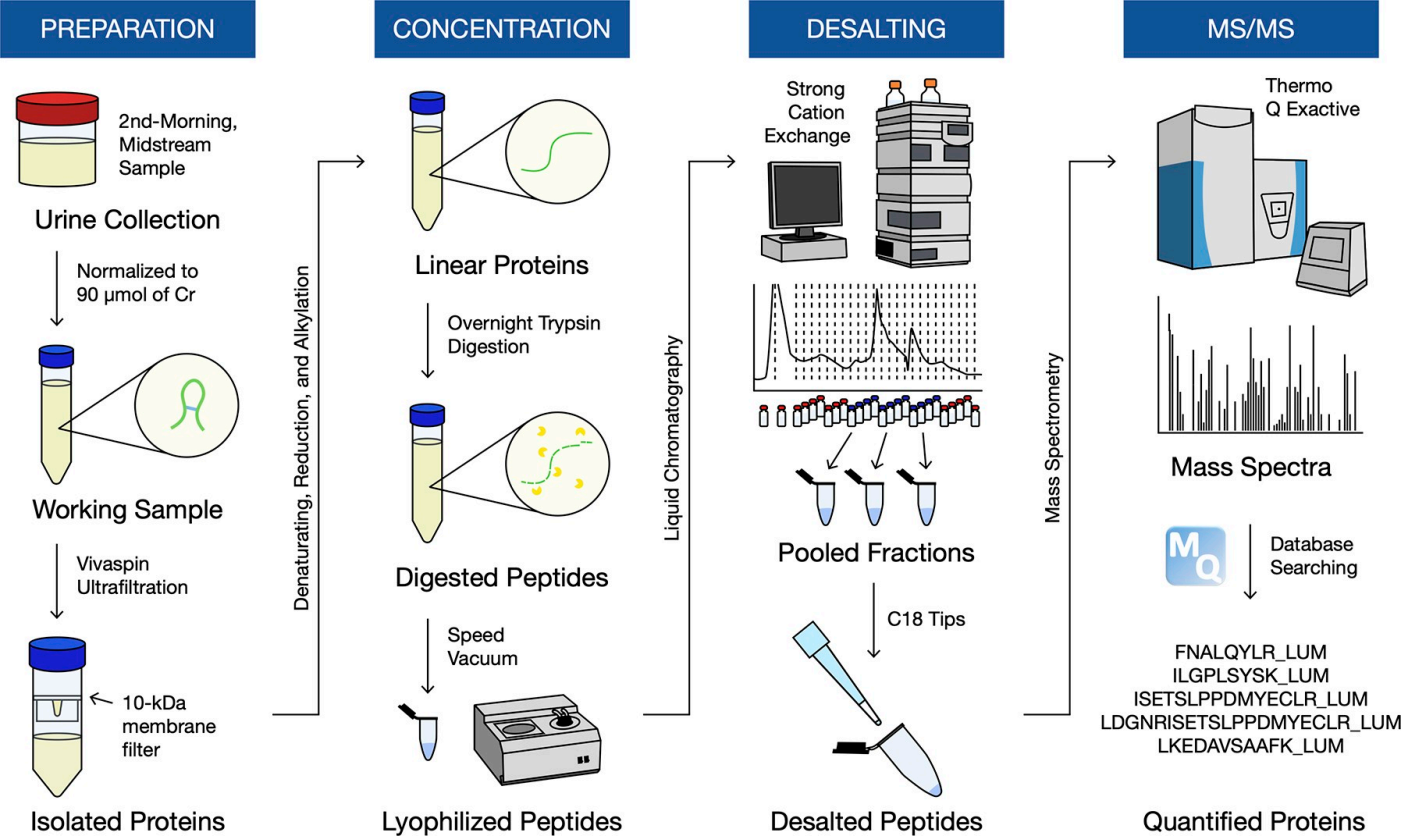
Workflow for the discovery-based urinary proteomics.

The workflow is summarized in Fig 1. After thawing, urines were vortexed and centrifuged at 1000 g for 10 minutes. To account for differences in hydration, we used volumes containing 90 μmol of urinary creatinine. Ammonium bicarbonate was added to increase alkalinity of samples (to a pH of 8) for later steps. We passed the urines through Vivaspin Centrifugal Concentrators (VivaProducts) with 10-kDa cut-off membranes to isolate the protein content. (The filtrate was analysed using urinary peptidomics [13].) Protein concentrations were measured in the retentate using the bicinchoninic acid (BCA) assay such that protein quantification was normalized by total protein amount. A total of 200 μg of protein was denatured with urea, reduced with dithiothreitol, alkylated with iodoacetamide, and digested overnight with trypsin. Proteolysis was terminated after 16 hours on the next day using formic acid. Peptides were subsequently speed-vacuumed to remove excess water content and reduce volumes to below 200 μL.

Next, we performed strong cation exchange high-performance liquid chromatography (SCX-HPLC). Peptides were loaded onto a PolySULFOETHYL ATM column (The Nest Group Incorporation) containing a hydrophilic, anionic polymer (poly-2-sulfoethyl aspartamide). We created a 60-minute fractionation method with a linear gradient of 0.26 M formic acid in 5% acetonitrile as the running buffer and 1 M ammonium formate added as the elution buffer. We combined 12 of the 26 fractions, which covered the largest area on the chromatogram, into 3 pooled fractions. Further desalting and concentration were carried out with Agilent Technologies Bond Elut OMIX C18 tips with a bed mass of 8 μg. Peptides were then eluted in 5 μL of 65% acetonitrile and diluted with 60 μL of 0.1% formic acid in pure mass spectrometry-grade water; 18 μL of which was injected into the mass spectrometer (~2 μg of total protein per injection).

Overall, three fractions per youth were analyzed over a 60-minute gradient on a Thermo Scientific EASY-nLC1000 system, coupled to a Thermo Scientific Q Exactive Plus hybrid quadrupole-orbitrap mass spectrometer using nano-electrospray ionization. They were first loaded onto a 3.3 cm C18 pre-analytical column (IntegraFrit capillary, New Objective; inner diameter of 150 μm; 5 μm bead size; Agilent Pursuit C18, Agilent Technologies) and then a C18 resolving analytical column with dimensions 15 cm x 75 μm ID (PicoTip emitter, 8 μm tip, New Objective Agilent Pursuit C18, 3 μm bead size). Data-dependent acquisition mode was used with full MS1 scans from 400–1500 m/z with a resolution of 70,000 and MS2 scans of the top 12 parent ions with a resolution of 17,500. Xcalibur software (v. 3.0.63; Thermo Fisher Scientific) was utilized to generate RAW files of each run. Mass spectrometry data have been deposited onto the ProteomeXchange Consortium via the PRIDE [15] partner repository with the dataset identifier PXD017213 (http://www.ebi.ac.uk/pride/archive/login).

### Mass spectrometry analysis

Raw data were analyzed by MaxQuant software (version 1.5.3.8) and were searched against the human Uniprot FASTA database (July 2016 version containing 42158 protein entries) using the built-in Andromeda search engine [16]. The false discovery rate was set to 1% using a revert database for both proteins and peptides with a minimum length of six amino acids. The digestion mode was specific for trypsin/P with a maximum of 2 missed cleavages. Cysteine carbamidomethylation was selected as a fixed modification; while methionine oxidation, proline oxidation, and N-terminal acetylation as variable modifications. Potential contaminants were allowed in the search and manually removed *post hoc*. The initial peptide tolerance was set to 20 ppm against a small ‘human-first-search’ database. The main search peptide mass tolerance was 4.5 ppm, and the fragment mass tolerance was set to 0.5 Da. Matching between runs was selected. Stabilized label-free quantification (LFQ) of proteins derived from extracted ion current information from razor and unique peptides with a minimum ratio count of 2. The fast LFQ option was selected with a minimum ratio count of 2, minimum of 3 neighbours, and average of 6 neighbours.

We analyzed the proteomic data using Perseus software (version 1.5.5.3) [17]. Reverse hits were first filtered out; non-human contaminants were manually checked and removed. We then examined the subset of proteins identified in 100% of urine samples to focus on the most robust changes in the proteome. Fast LFQ intensities were log(*x*) transformed to approximate a normal distribution. Differential LFQ intensities between groups were then determined using the independent t-test (*P* < 0.05), followed by BH adjustment (*Q* < 0.05).

### Bioinformatic analyses

The Human Protein Atlas was searched to determine tissue origins of urinary proteins [18]. Plots were created with R software. For the heatmap analysis, we converted peptide intensities into z-scores and performed Euclidean hierarchical clustering. We also searched the pathDIP tool (version 4.0.21.2; http://ophid.utoronto.ca/pathDIP/) [19] using all sources, KEGG BRITE and Pathway Mapper tools (version 4.1; https://www.genome.jp/kegg/) [20], and Reactome (version 71; https://reactome.org/)) [21, 22]. The Integrated Interactions Database (IID, version 2018–11; http://iid.ophid.utoronto.ca/) was used to retrieve experimentally-proven and predicted protein-protein interaction data [23].

### Enzyme-Linked Immunosorbent Assay (ELISA)

Urinary protein concentrations were measured in duplicate with commercial human ELISA kits: from R&D Systems, cluster of differentiation 14 (DY383), lumican (DY2846), and vascular cell adhesion molecule 1 (DY809); and from Raybiotech, hexosaminidase A (ELH-HEXA-1). We also purchased and used the R&D Systems Ancillary Reagent Kit 2 (DY008) in conjunction with the aforementioned R&D Systems Duoset ELISA kits. Frozen urine samples were thawed and kept at 4°C until further processing, centrifuged at 2000 g for 5 minutes, and diluted in the appropriate reagent diluent supplied by the kit. All plates were read by spectrophotometry at 450 nm, and label subtraction was carried out at 540 nm using EnVision 2103 Multilabel Reader (Perkin Elmer, Waltham, MA, USA). Urinary concentrations were determined from standard curves according to specific kit instructions and were adjusted for urinary creatinine concentrations. Differential peptide excretion was determined using the Mann-Whitney test (*P* < 0.05) in the validation cohort.

## Results

### Characterization of urinary proteome of early type 1 diabetes

**Fig 2:**
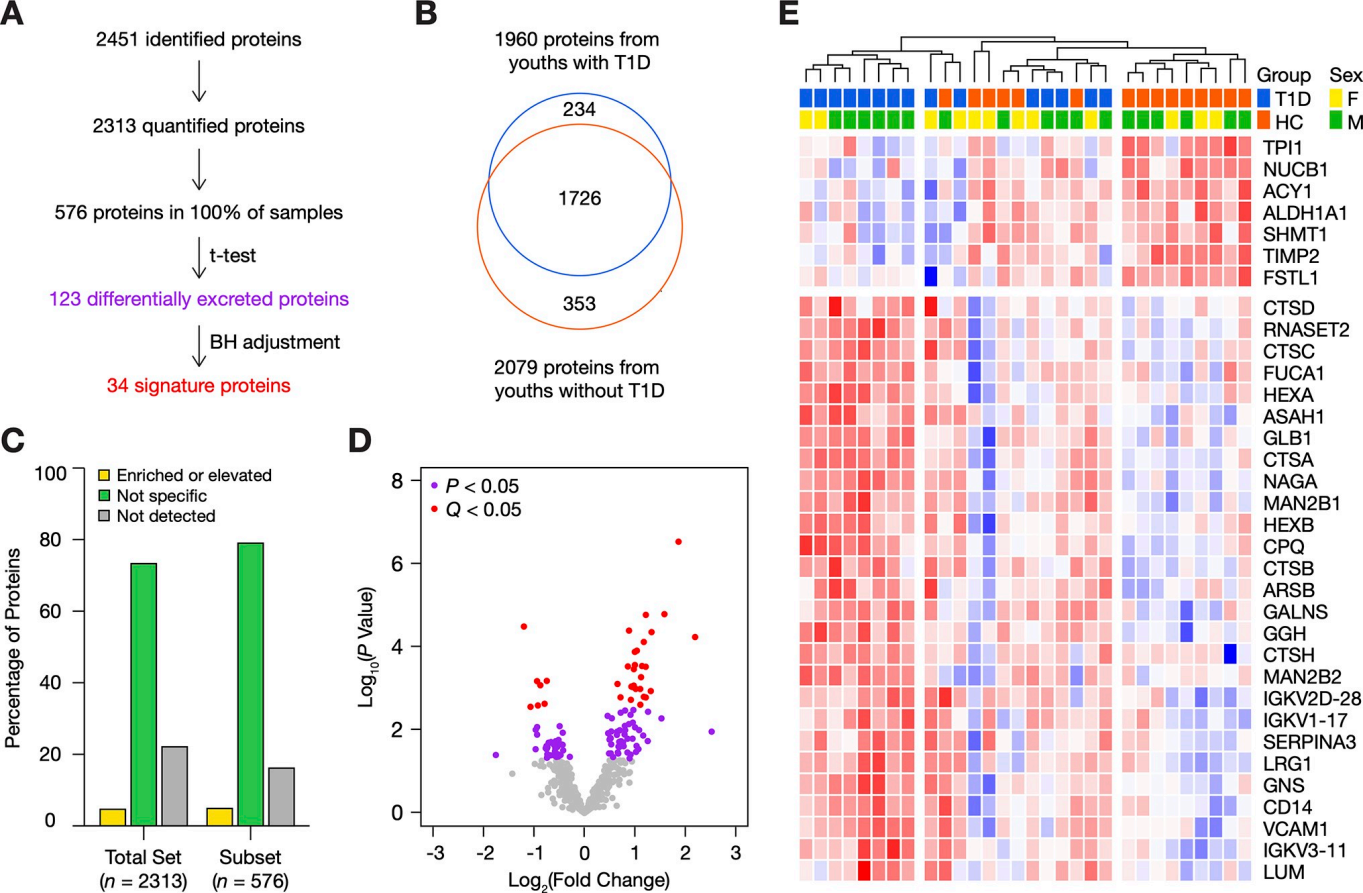
Characterization of the urinary proteomes of 15 youths with type 1 diabetes and 15 non-diabetic peers. (A) Flow diagram for the identification of the urinary signature of early type 1 diabetes. (B) Venn diagram of the 2313 quantified urinary proteins. (C) Kidney tissue origins of the total proteome and the subset of 576 proteins using the Human Protein Atlas. (D) Volcano plot of the 576 proteins found in all samples. A total of 123 proteins was differentially excreted (independent two-sample Student’s t-test, *P* < 0.05, purple); and 34 survived BH adjustment (*Q* < 0.05, red). Higher fold changes indicate that protein intensities were higher in type 1 diabetes. (E) Heatmap representation of the 34 signature proteins with unsupervised clustering of samples. Log-transformed intensities were converted into *z*-scores. Scores were coloured on a blue (low) to red (high) gradient. F, female; HC, non-diabetic youths; M, male; T1D, youths with type 1 diabetes.

A summary of the proteomic workflow is illustrated in Fig 1. Overall, we identified 2451 urinary proteins from 30 otherwise healthy youths with and without type 1 diabetes (Fig 2A). A total of 2313 proteins was quantified (S1 Table): 1960 in youths with type 1 diabetes, 2079 in youths without type 1 diabetes, and 1726 in both groups (Fig 2B). There were 234 proteins that exclusively derived from the diabetic group; however, none were quantified in more than 3 urine samples. Similarly, the 353 proteins that were only found in the non-diabetic group were quantified in a small minority of samples. To examine the most robust changes in the urinary proteome, we focused on a subset of 576 proteins (S2 Table), which were quantified in every urine sample. According to the Human Protein Atlas [18], more than 80% of proteins can be found in the kidney in an enriched/elevated (5%) or non-specific (79%) pattern (Fig 2C). We then compared protein intensities between groups and identified 123 differentially excreted proteins (*P* < 0.05) (Fig 2D). Thirty-four proteins remained significant following BH adjustment (*Q* < 0.05) (Table 2). The heatmap analysis of these 34 proteins highlighted two distinct clusters of youths based on diabetes status, although segregation was incomplete (Fig 2E). Urinary excretion of the signature proteins appears to be coordinated, as the majority of signature proteins were strongly correlated with one another (|*r|* > 0.6) (S1 Fig).

**Table 2:** Summary of urinary proteins significantly altered in early type 1 diabetes.

Protein Name (Gene)	UniProt Number	Fold Change	*P*	*Q*	Fraction of Unique Peptides	Sequence Coverage (%)
**Aminoacylase-1 (ACY1)**	Q03154	0.44	0.0000	0.0047	23/23	67
**Serine hydroxymethyltransferase (SHMT1)**	P34896	0.48	0.0028	0.0471	19/19	55
**Metalloproteinase inhibitor 2 (TIMP2)**	P16035	0.52	0.0007	0.0214	8/8	36
**Triosephosphate isomerase (TPI1)**	P60174	0.53	0.0026	0.0451	23/23	89
**Follistatin-related protein 1 (FSTL1)**	Q12841	0.55	0.0008	0.0242	13/13	44
**Retinal dehydrogenase 1 (ALDH1A1)**	P00352	0.58	0.0024	0.0437	23/25	65
**Nucleobindin-1 (NUCB1)**	Q02818	0.60	0.0007	0.0224	34/34	63
**Acid ceramidase (ASAH1)**	Q13510	1.58	0.0008	0.0237	24/24	58
**Beta-hexosaminidase subunit alpha (HEXA)**	P06865	1.64	0.0016	0.0338	22/22	41
**Cathepsin D (CTSD)**	P07339	1.82	0.0003	0.0131	29/29	66
**Arylsulfatase B (ARSB)**	P15848	1.85	0.0000	0.0047	15/15	31
**Tissue alpha-L-fucosidase (FUCA1)**	P04066	1.89	0.0019	0.0362	12/12	31
**Ribonuclease T2 (RNASET2)**	O00584	1.91	0.0009	0.0237	13/13	45
**Alpha-1-antichymotrypsin (SERPINA3)**	P01011	1.94	0.0009	0.0232	29/29	61
**Cathepsin H (CTSH)**	P09668	1.97	0.0009	0.0235	16/16	61
**N-acetylglucosamine-6-sulfatase (GNS)**	P15586	1.97	0.0003	0.0132	23/23	41
**Beta-hexosaminidase subunit beta (HEXB)**	P07686	2.00	0.0001	0.0076	27/27	49
**Beta-galactosidase (GLB1)**	P16278	2.00	0.0003	0.0143	27/27	40
**Ig kappa chain V-III region VG (IGKV3-11)**	P04433	2.03	0.0010	0.0247	4/4	57
**Lysosomal protective protein (CTSA)**	P10619	2.06	0.0001	0.0079	17/17	40
**Cathepsin B (CTSB)**	P07858	2.15	0.0010	0.0238	21/21	62
**Lumican (LUM)**	P51884	2.16	0.0025	0.0445	20/20	45
**Ig kappa chain V-I region WEA (IGKV1-17)**	P01610	2.18	0.0005	0.0194	2/4	37
**Gamma-glutamyl hydrolase (GGH)**	Q92820	2.21	0.0003	0.0139	19/19	46
**Ig kappa chain V-II region TEW (IGKV2D-28)**	P01617	2.26	0.0016	0.0341	1/3	39
**N-acetylgalactosamine-6-sulfatase (GALNS)**	P34059	2.26	0.0001	0.0055	23/23	57
**Epididymis-specific alpha-mannosidase (MAN2B2)**	Q9Y2E5	2.32	0.0003	0.0124	32/32	38
**Monocyte differentiation antigen CD14**	P08571	2.33	0.0017	0.0328	21/21	71
**Carboxypeptidase Q (CPQ)**	Q9Y646	2.33	0.0000	0.0032	19/19	49
**Vascular cell adhesion protein 1 (VCAM1)**	P19320	2.49	0.0012	0.0257	23/23	39
**Dipeptidyl peptidase 1 (CTSC)**	P53634	2.52	0.0000	0.0042	21/21	45
**Alpha-N-acetylgalactosaminidase (NAGA)**	P17050	3.00	0.0000	0.0047	16/16	39
**Lysosomal alpha-mannosidase (MAN2B1)**	O00754	3.65	0.0000	0.0003	30/30	39
**Leucine-rich alpha-2-glycoprotein (LRG1)**	P02750	4.58	0.0001	0.0048	19/19	57

Fold change represents the ratio of the median label-free quantification (LFQ) protein intensity of the diabetic group to the median value of the non-diabetic group. *P* values were determined using the Student t-test and then corrected with the Benjamini-Hochberg adjustment (*Q*). The number of unique peptides for each protein is shown as a fraction over the total number of peptides.

### Lysosomal enzymes dominate urinary protein signature of early diabetes

We first used the pathDIP tool [19] (version 4.0.21.2) as a broad survey of the enriched pathways associated with the 34 signature proteins (S3 Table). Even though the tool collates data from 24 sources, pathways from KEGG and Reactome databases were most commonly represented in the enriched set (BH-adjusted, *Q* < 0.05).

**Fig 3:**
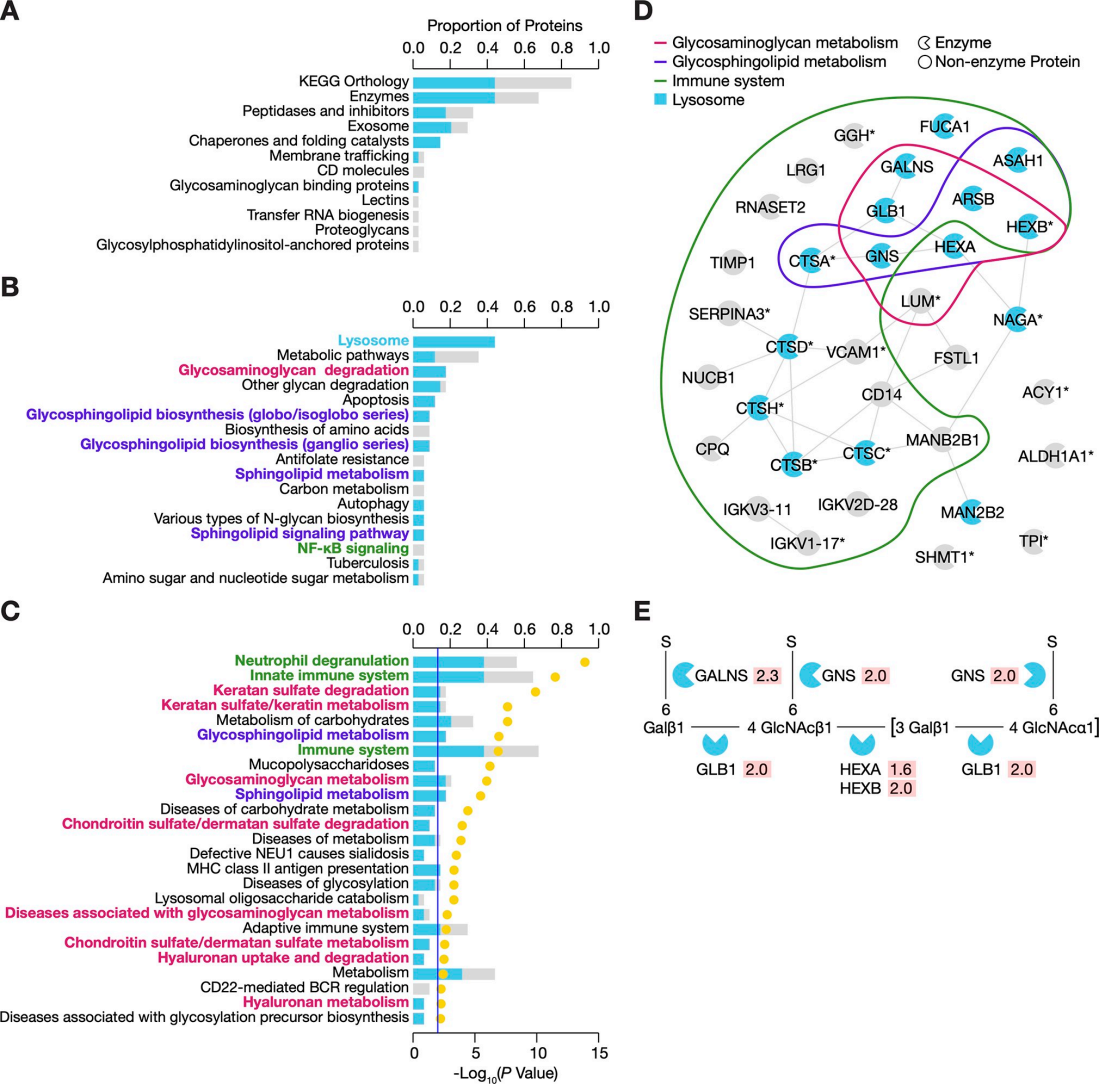
Lysosomal enzymes involved in glycosaminoglycan metabolism and the immune system dominated the urinary signature of early type 1 diabetes. (A) Top KEGG functional hierarchies associated with the signature proteins. Stacked bars indicate the proportions of lysosomal (blue) and non-lysosomal (grey) proteins in the urinary signature. (B) Top KEGG pathways associated with two or more signature proteins. Stacked bars indicate the proportions of lysosomal (blue) and non-lysosomal (grey) proteins in the urinary signature. (C) Enriched Reactome pathways associated with signature proteins (*P* < 0.05). Stacked bars indicate the proportion of lysosomal (blue) and non-lysosomal (grey) proteins in the urinary signature and correspond with the left axis. Dots represent statistical significance and correspond with the right axis. (D) Protein-protein interaction network of urinary signature proteins. Clusters highlight protein involvement in glycosaminoglycan metabolism, glycosphingolipid metabolism, and immune system. Blue colour indicates a protein associated with the lysosome. (E) Visualization of the reactions and enzymes involved in keratan sulfate degradation. Fold changes (relative to youths without diabetes) are shown on a red background next to each enzyme.

We then searched the KEGG Pathway and BRITE Mapper tools [20] to identify all of the pathways and functional hierarchies associated with the top 34 proteins (S4 Table). Five proteins were not identified in the KEGG Orthology. Of the 29 proteins searched, 23 were classified as enzymes (Fig 3A). Fifteen enzymes were mapped to the lysosome (Fig 3B), which included five members of the cathepsin protease family (CTSA, CTSB, CTSC, CTSD, and CTSH), five glycosidases (tissue alpha-L-fucosidase, FUCA1; β-galactosidase, GLB1; beta-hexosaminidase subunit alpha and beta, HEXA and HEXB; lysosomal alpha-mannosidase, MAN2B1; and alpha-*N*-acetylgalactosaminidase, NAGA), three sulfatases (arylsulfatase B, ARSB; *N*-acetylgalactosamine-6-sulfatase, GALNS; and *N*-acetylglucosamine-6-sulfatase, GNS), and acid ceramidase (ASAH1). Other notable pathways included metabolism, glycosaminoglycan degradation, other glycan degradation, glycosphingolipid biosynthesis (ganglio and globo series), and apoptosis.

We also searched the Reactome pathway database (version 71) [21, 22] to replicate the KEGG findings and to focus on specific pathways and reactions. Two proteins, carboxypeptidase Q (CPQ) and NAGA, were not identified in the Reactome database and were thus not included in this analysis (S5 Table). A total of 154 pathways were associated with at least one protein. Seventy-nine pathways were significant enriched (*P* < 0.05); twenty-five remained statistically significant after BH correction (*Q* < 0.01) (Fig 3C). The top three pathways were neutrophil degranulation, innate immune system, and keratan sulfate degradation.

By searching both databases, we were able to highlight several similarities. Notably, the lysosomal enzymes are involved in glycosaminoglycan metabolism, glycosphingolipid metabolism, and the immune system, namely neutrophil degranulation. Using the Integrated Interactions Database [23], we demonstrated that these signature proteins may interact with one another within these pathways (Fig 3D). Interestingly, all enzymes involved in keratan sulfate degradation (ie., GALNS, GLB1, GNS, HEXA, and HEXB) were elevated in urines from youths with type 1 diabetes, compared to non-diabetic youths (Fig 3E). Furthermore, urinary excretion of these five enzymes significantly correlated with one another with Pearson coefficients ranging between 0.61 to 0.80 (*P* ≤ 0.0002) (S2A Fig). In addition, urinary excretion of lumican, a core protein of keratan sulfate proteoglycan, was higher in diabetes with a fold change of 2.16 (*Q* = 0.0445) (Table 2). Lumican was most strongly correlated with GNS (*r* = 0.63, *P* = 0.0002), GALNS (*r* = 0.54, *P* = 0.0004), and HEXA (*r* = 0.52, *P* = 0.0030) (S2A Fig).

### Internal validation

**Fig 4:**
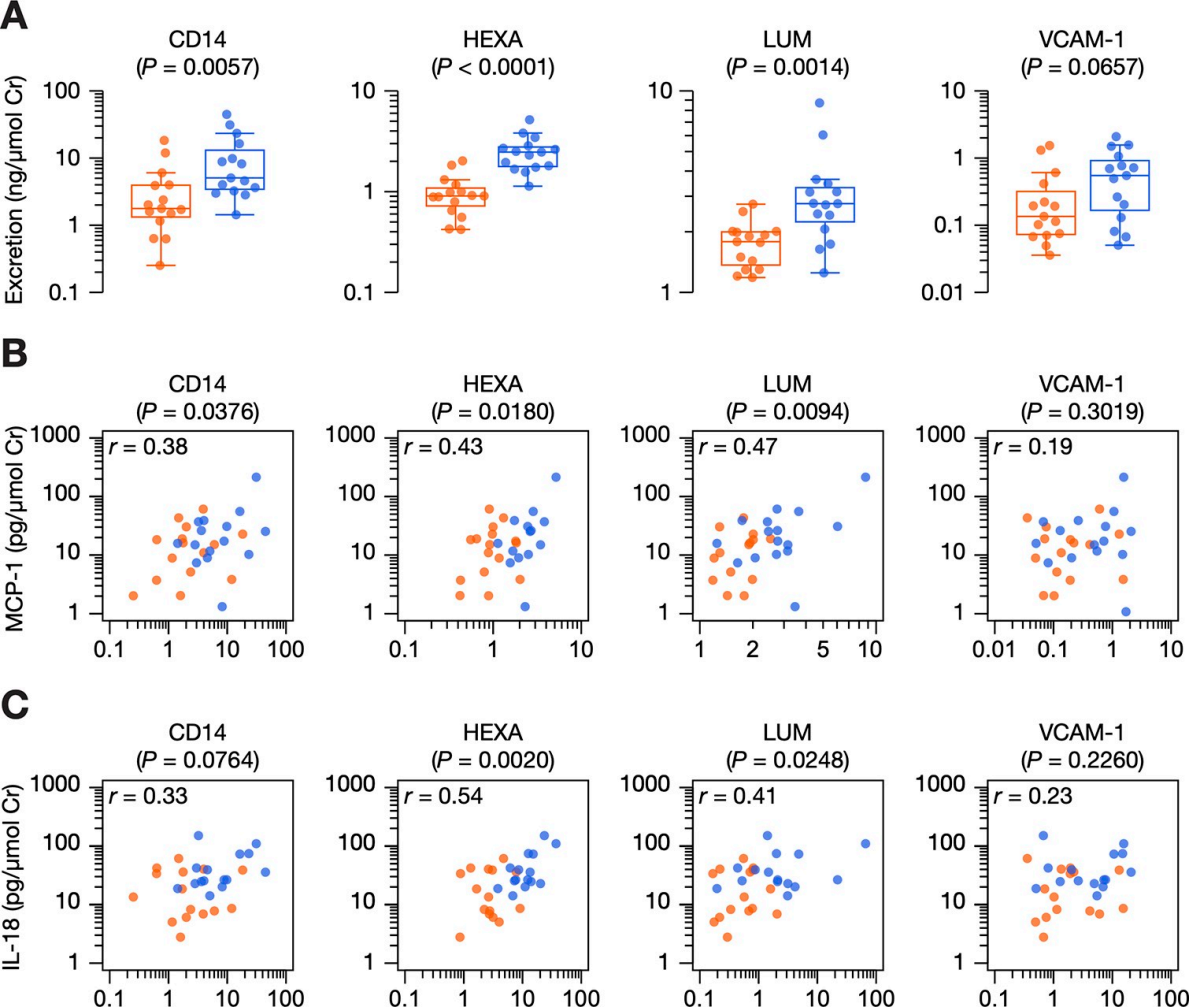
Internal validation of four proteins in a second, independent cohort. Individual data points are shown for the 15 youths with diabetes (blue) and 15 youths without diabetes (orange). (A) Urinary excretion of four proteins selected for validation by enzyme-linked immunosorbent assay. *P* values, determined by the Mann-Whitney test, are shown for each protein. (B) Pearson correlations between log-transformed urinary excretion of four proteins and that of monocyte chemoattractant protein-1 (MCP-1). (C) Pearson correlations between log-transformed urinary excretion of four proteins and that of interleukin-18 (IL-18). CD14, monocyte differentiation antigen CD14; HEXA, beta-hexosaminidase subunit alpha; HC, non-diabetic youths; LUM, lumican; T1D, youths with type 1 diabetes; VCAM-1, vascular adhesion molecule-1.

We selected four proteins for internal validation in a second, independent cohort of youths with and without type 1 diabetes on the basis of: 1) availability of a commercial enzyme-linked immunosorbent assay kit; 2) a role in keratan sulfate biology and degradation; and 3) a role in innate immunity. Increased urinary excretion of CD14 (*P* = 0.0057), HEXA (*P* < 0.0001), and lumican (*P* = 0.0014) was replicated in a second cohort of youths with and without type 1 diabetes (Fig 4A). Urinary excretion of VCAM-1 was also higher in diabetes, but it did not reach statistical significance (*P* = 0.0675). These four proteins were significantly correlated with one another with Pearson coefficients ranging between 0.47 and 0.81 (*P* ≤ 0.0083) (S2B Fig). The fold changes and Pearson coefficients were similar to those observed in the discovery cohort (Table 2, S2C Fig).

We next examined whether these four proteins may be associated with an early pro-inflammatory signal in diabetes. In a previous study [13], we had selected eight urinary cytokines and chemokines and demonstrated that interleukin (IL)-6, IL-8, IL-18, interferon gamma-induced protein 10 (IP-10), monocyte chemoattractant protein-1 (MCP-1), and macrophage inflammatory protein 1-beta (MIP1B) could be measured in urine from youths with and without diabetes. Although urinary excretion of MCP-1 did not appear to be significantly different between groups [13], it correlated with urinary excretion of CD14 (*r* = 0.38, *P* = 0.0376), HEXA (*r* = 0.43, *P* = 0.0180), and lumican (*r* = 0.47, *P* = 0.0094) (Fig 4B; S2B Fig). Interestingly, HEXA also correlated with IL-18 (*r* = 0.54, *P* = 0.0020) and IP-10 (*r* = 0.54, *P* = 0.0067) (Fig 4C). A complete summary of correlations between protein and cytokine/chemokine can be found in S2B Fig.

### Comparison with other studies

As an additional validation step, we compared our findings to other similarly designed studies. More specifically, we searched and reviewed the PubMed database and PRIDE Archive repository for studies that examined the urinary proteomics of otherwise healthy populations with diabetes using the following search terms and their variations: “urine”, “proteomic”, “diabetes”, “early”, and “human”. To ensure that urinary protein signatures of each study reflected the early mechanisms of injury or renal compensation, we excluded studies that included individuals with clinical diabetic kidney disease (e.g., microalbuminuria, proteinuria, decline in glomerular filtration rate) and other complications (e.g., cardiovascular disease, hypertension). A total of three relevant studies met these criteria [24–26].

**Fig 5:**
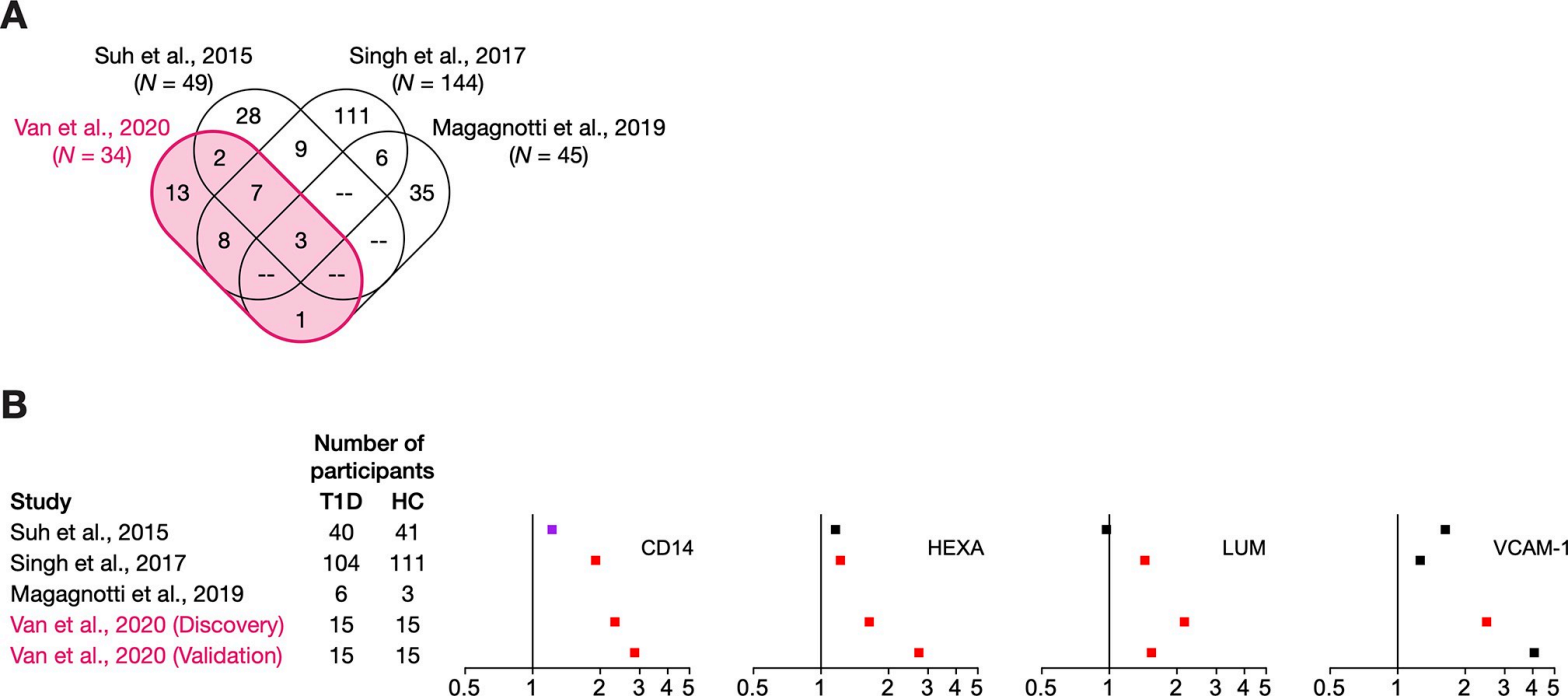
Comparison of current findings with the literature. (A) Venn diagram of the urinary signature proteins from our current study (highlighted in red) and three other proteomic studies of early diabetes. Signature proteins were defined on the basis of statistical significance after Benjamini-Hochberg adjustment (*Q* < 0.05) for three studies [24, 25], including our analysis, or on the basis of a combination of factors (|fold change| > 1.6, *P* < 0.06) for the study by Magagnotti and colleagues [26]. (B) Forest plots of fold changes of urinary excretion of the four proteins selected for validation. Fold change is calculated as a ratio of the median excretion of youths with diabetes to that of youths without diabetes. Colours delineate proteins that belong to the urinary signature of the indicated study (red, *Q* < 0.05); proteins that were differentially excreted but lost statistical significance after Benjamini-Hochberg adjustment (purple, *P* < 0.05); and proteins that failed to reach statistical significance before and after adjustment (black, *P* ≥ 0.05). CD14, monocyte differentiation antigen CD14; HEXA, beta-hexosaminidase subunit alpha; HC, non-diabetic youths; LUM, lumican; T1D, youths with type 1 diabetes; VCAM-1, vascular adhesion molecule-1.

We then retrieved and collated publicly available data to define the urinary protein signature for each of the three studies. For two studies [24, 25], we applied the same criteria as our study for significant differential excretion on the basis of BH adjustment (*Q* < 0.05). For one study [26], we included all 45 proteins identified from the 61 spots that had an absolute fold change above 1.6 and a *P* value below 0.06 after hierarchical clustering to identify the most radically different subset of youths with very early type 1 diabetes (duration of less than one year) from youths without diabetes. We found that 21 proteins from our signature were also differentially excreted in at least one of the three other proteomics studies of early diabetes (Fig 5A; S6 Table). Notably, three proteins (CPQ, CTSB, and GNS) were common among all four urinary signatures, and they were consistently excreted at higher rates in diabetes. Of the 13 proteins that were uniquely associated with our signature for early diabetes, we note that five had also been differentially excreted in the other studies (*P* < 0.05) but statistical significance was lost after multiple testing correction (*Q* > 0.05), suggesting that they may be differentially excreted in diabetes, but have a smaller overall effect size.

Forest plots were subsequently created to visualize fold changes and the degree of statistical significance of the four proteins selected for validation (Fig 5B). These proteins (CD14, HEXA, LUM, and VCAM-1) were not among the differentially excreted proteins identified by Magagnotti and colleagues. Urinary excretion of CD14, HEXA, and LUM was significantly elevated in early diabetes as reported by Singh and colleagues [24], but was variable in the study by Suh and colleagues [25]. We also examined the other proteins involved in keratan sulfate biology and degradation in the external studies and found that increased excretion of GALNS, GLB1, GNS, and HEXB was also reported by at least one other study (S3 Fig).

We also searched the Nephroseq v5 database (www.nephroseq.org, March 2020, University of Michigan, Ann Arbor, MI) for renal expression data of the urinary signature proteins. These transcriptomic datasets allow for a more comprehensive examination of genes dysregulated in various renal pathologies. Of the 34 signature proteins, 32 were differentially expressed with absolute fold changes above 2 (S7 Table). Expression of more than half of our signature proteins was markedly altered in datasets characterizing chronic kidney disease (with 27 proteins), diabetic nephropathy (with 21 proteins), and lupus (with 18 proteins). However, the direction of fold change was highly discordant in chronic kidney disease compared to our urinary expression levels, as 15 out of the 27 proteins with altered mRNA expression were mismatched. In contrast, we noted that only three proteins in diabetes and two in lupus were discordant. Of the 4 proteins selected for validation, we observed that CD14, lumican, and VCAM-1 are overexpressed in subsets characterizing diabetic nephropathy, focal segmental glomerulosclerosis, and lupus; however, our observed increases in HEXA urinary excretion was not reflected at the tissue level in Nephroseq as only two datasets reported significantly altered expression in opposing directions. Nevertheless, the overall findings demonstrate that mRNA levels of our signature proteins are altered at the tissue level in various etiologies of chronic kidney disease.

## Discussion

Chronic exposure to high glucose results in several compensatory and maladaptive responses in the kidney. However, the mechanisms responsible for initiating diabetic kidney disease are poorly understood. This gap in knowledge may be, at least in part, responsible for the lack of effective treatment strategies that prevent, cure, or reverse diabetic kidney disease. In this study, our goal was to examine the early effects of chronic hyperglycemia on the diabetic kidney. Using a discovery-based proteomic analysis of urine samples from otherwise healthy youths with and without type 1 diabetes, we identified a urinary signature of 34 proteins dominated by lysosomal enzymes. Additionally, these proteins are involved in neutrophil degranulation, the innate immunity, and keratan sulfate degradation. Increased urinary excretion of CD14, HEXA, and lumican was validated using ELISA assays in a second cohort. These candidates were also identified in other proteomic studies as potential indicators of early type 1 diabetes. Our findings suggest that lysosomal enzymes, glycosaminoglycan metabolism, and innate immunity may be perturbed in early diabetes before classic indications of clinical injury.

Urinary proteomics has been extensively employed to identify markers of progressive diabetic kidney disease. Much of the attention has been placed on later stages of disease, in which some degree of renal impairment is present [11]. Recent studies have instead shifted the spotlight on early diabetes before the development of microalbuminuria or glomerular filtration rate decline [24–26]. Notably, Suh and colleagues examined the urinary proteomes of youths with type 1 diabetes and included their non-diabetic siblings as healthy controls using filter-aided sample preparation [25]. Similar to our findings, lysosomal enzymes had emerged as important constituents in their urinary signature and were thought to indicate early inflammation in the renal vasculature [25]. Singh and colleagues also reported that youths with type 1 diabetes excreted higher amounts of lumican, CD14, and various lysosomal proteins such as ASAH1, CTSD, and NAGA compared to their non-diabetic siblings [24]. The authors speculated that these proteins may reflect changes in extracellular matrix [24]. In a multi-omics study, Magagnotti and colleagues first performed gel-based proteomic profiling of youths within 1 year of diagnosis of type 1 diabetes to establish an early predictive signature of kidney dysfunction [26]. Two lysosomal enzymes, ARSA and GLB1, were highlighted as part of the predictive profile for diabetic nephropathy. Various ceramides were subsequently examined by lipidomic profiling as downstream metabolites of ARSA and GLB1. The authors demonstrated that urinary levels of the lysosomal enzymes and ceramides were markedly increased in diabetic nephropathy. Interestingly, lysosomal enzymes have also been associated with other diabetic complications such as retinopathy, neurodegeneration, and microangiopathy [27–29]. Our analyses using Nephroseq datasets suggest that these enzymes are also overexpressed in renal tissues from individuals with chronic kidney disease, diabetic nephropathy, and lupus nephritis.

Even though the individual proteins differ between proteomic studies of early diabetes, the gene ontology terms that were overrepresented by the three published urinary signatures converge onto lysosomal enzymes [24–26]. According to our pathway analyses, the lysosomal enzymes are linked to neutrophil degranulation, innate immune system, and keratan sulfate degradation. Neutrophils are recruited to the site of injury as part of the early inflammatory phase of wound healing. Upon differentiation, these cells form granules containing hydrolytic enzymes and extracellular matrix components, which are functionally similar to lysosomes [30, 31]. Granules can mobilize to and fuse with the plasma membrane to release its secretory contents into the extracellular space and present its membrane contents onto the cell surface via exocytosis. Degranulation is one of the three main mechanisms by which neutrophils help clear pathogens [32]. Increased granule release has also been implicated in several autoimmune diseases such as asthma, lupus, and rheumatoid arthritis [33–35]. Although inflammation is often associated later stages of diabetic kidney disease, there is growing support for its early activation before clinical manifestations of kidney injury [36, 37]. We thus posit that increased urinary excretion of lysosomal enzymes may reflect an early activation of inflammation in response to chronic hyperglycemia.

Renal inflammation is a critical hallmark of diabetic kidney disease initiation and progression. Chronic hyperglycemia is known to disrupt the metabolic milieu inside the cell. The cell adapts by redirecting excess intracellular glucose into one of four hypothesized pathways: the polyol pathway, the formation of advanced glycation end-products, the protein kinase C signaling pathway, and the hexosamine pathway—all of which contribute to or reflect an increased production of reactive oxygen species. As a result, the primary initiating event in the development of diabetic complications is injury from oxidative stress [38–40]. Recent studies have also implicated the sterile, pathogen-free activation of toll-like receptors (TLRs), NFκB signaling, and the inflammasome, as part of the unresolved inflammatory response in the diabetic kidney [41–44]. In other words, TLR signalling can be triggered by damaged-associated molecular patterns (DAMPs) that are released by injured or stressed kidney cells [44, 45], thereby perpetuating the cycle of injury-inflammation response.

Keratan sulfate degradation is a natural turnover process that occurs in the lysosome. Remarkably, our urinary signature for early diabetes includes a core protein of the keratan sulfate proteoglycan (lumican) and the complete set of putative enzymes that break down keratan sulfate (GALNS, GLB1, GNS, HEXA, and HEXB), which were all excreted to a greater extent by youths with type 1 diabetes, compared to their non-diabetic peers and were strongly correlated with one another. Keratan sulfates consist of repeating disaccharide units of *N*-acetylglucosamine and galactose (GlcNAc-Gal) [46]. As proteoglycans, keratan sulfates attach to either asparagine (*N*-linked) or serine/threonine (*O*-linked) residues of core proteins such as lumican, aggrecan, and fibromodulin. The glycosidic bonds are severed by GLB1, HEXA, HEXB to release the sugar monomers; the sulfates, by GALNS and GNS. The degree of sulfation varies and plays an important role in adhesion, as macrophages preferentially bind to lumican with minimally sulfated keratan sulfate chains over intact forms [47]. Furthermore, the proteoglycan modulates the innate immune response, by interacting with the TLR4 co-receptor, CD14 [48, 49] and by facilitating neutrophil extravasation [50, 51]. Interestingly, TLR4 activation was highlighted as a potential mechanism in a genome-wide association study of diabetic kidney disease [52], linking keratan sulfate biology to inflammation. Lumican also regulates collagen fibril assembly and function [53] and contributes to wound healing [54, 55]. Although keratan sulfate proteoglycans are expressed throughout the body, they have been largely described in the cornea, brain, bone, and cartilage [46]. Previous studies have identified differences in lumican-based proteoglycan in healthy and diabetic kidney. In tumour-free tissues from otherwise healthy individuals undergoing nephrectomies for renal cell carcinoma, lumican-based proteoglycans were predominantly detected in the tubulointerstitium, deriving from peritubular mesenchymal cells, with a lower expression in the mesangial matrix of the glomerular compartments; in diabetic tissues, a marked increase in tubular expression was observed, which was not paralleled in glomeruli until advanced diabetic kidney disease was established [56, 57]. Additional experimental work is required to clarify whether increased urinary excretion of lysosomal enzymes may reflect a loss of enzymatic turnover activity in the kidney, thereby explaining the observed overexpression of lumican proteoglycans in diabetic kidney tissues.

Our study provides robust data that are consistent with and affirm previous findings, in which lysosomal perturbations may be at the forefront of the early diabetic kidney response. Dysregulated pathways in keratan sulfate metabolism have not previously been linked to early diabetes and represent a novel observation. We were also able to replicate increased urinary excretion of a select number of proteins in our internal validation cohort. Additionally, we performed an external validation by comparing our findings to other proteomic studies, despite differences in sample preparation and methodologies. Finally, our raw data and complete protein lists have been made publicly available.

Our study has limitations. First, we conducted a cross-sectional study and therefore cannot draw conclusions about cause-effect relationships between diabetes and changes in the urinary proteome. Our study nevertheless provides important preliminary data for future studies that will assess the clinical utility of these differentially excreted proteins in larger, more heterogeneous populations that include individuals with type 2 diabetes and at varying stages of diabetic kidney disease. Second, additional experimental work is required to examine the biology pertaining to keratan sulfate proteoglycans and lysosomal enzyme activity in the diabetic kidney before the onset of clinically-detectable disease. Advancements in single-cell transcriptomics and proteomics will enable us to pinpoint the exact origins of these urinary signature proteins and their potential localizations in nephron segments. Furthermore, *in vitro* and *in vivo* studies could also clarify whether chronic hyperglycemic conditions promote the lysosomal permeabilization and subsequent release of enzyme from kidneys and immune cells into urine and whether the shedding of lysosomal enzymes is associated with decreased intrarenal enzymatic activity and reduced turnover of proteoglycans. Third, our validation efforts were constrained by the limited availability of specific antibodies and ELISA kits, especially ones that have been designed and optimized for urinary measurements. A future goal would be to develop multiplexed targeted assays using selective or parallel reaction monitoring so that we can validate a more comprehensive set of lysosomal enzymes and protein targets.

## Conclusions

In conclusion, we identified a urinary proteomic signature for early type 1 diabetes, of which lysosomal enzymes were major constituents. Our present findings suggest that lysosomal enzymes, neutrophil degranulation, innate immunity, and keratan sulfate degradation may be involved in the early kidney response to hyperglycemia. Additional studies are needed to validate protein excretion in larger and broader populations and to elucidate the impact of lysosomal enzymes and keratan sulfate degradation in the diabetic kidney.

## Supporting information

S1 Table List of the 2313 quantified urinary proteins from the discovery cohort. (XLSX)

S2 Table List of the 576 urinary proteins found in every urine sample from the discovery cohort. (XLSX)

S3 Table Results from the pathDIP database using the 34 signature proteins. (XLSX)

S4 Table Enriched KEGG pathways and functional hierarchies associated with the 34 signature proteins. (XLSX)

S5 Table Enriched Reactome pathways associated with the 34 signature proteins. (XLSX)

S6 Table Fold changes of the 34 signature proteins reported by other similarly designed published studies. (XLSX)

S7 Table Fold Changes (FC) of the 34 signature proteins as reported by Nephroseq transcriptomic datasets comparing renal expression in individuals with varying kidney diseases to living or deceased donors. (XLSX)

S1 Fig Correlogram of urinary excretion of the 34 signature proteins from the discovery cohort. Pearson correlations of log-transformed protein label-free quantification (LFQ) intensities are shown before the protein header. *P* values are shown above the header.
(DOCX)

S2 Fig Correlograms of urinary excretion of specific signature proteins. Pearson correlations of log-transformed protein intensities are shown before the protein header. *P* values are shown above the header. (A) Sub-analysis of the five enzymes associated with keratan sulfate degradation and lumican, a core protein of keratan sulfate. Data is from the discovery cohort. (B) Sub-analysis of the four proteins selected for internal validation and six urinary cytokines/chemokines. Data is from the validation cohort. (C) Sub-analysis of the four proteins selected for internal validation. Data is from the discovery cohort.
(DOCX)

S3 Fig Forest plots of fold changes of urinary excretion of the five enzymes associated with keratan sulfate degradation and lumican, a core protein of keratan sulfate proteoglycan. Fold change is calculated as a ratio of the median excretion of youths with diabetes to that of youths without diabetes. Colours delineate proteins that belong to the urinary signature of the indicated study (red, *Q* < 0.05); proteins that were differentially excreted but lost statistical significance after Benjamini-Hochberg adjustment (purple, *P* < 0.05); and proteins that failed to reach statistical significance before and after adjustment (black, *P* ≥ 0.05).
(DOCX)
