# Supplementary material for: Urinary proteomics links keratan sulfate degradation and lysosomal enzymes to early type 1 diabetes
Source: PLoS One. 2020 May 26;15(5):e0233639. doi: 10.1371/journal.pone.0233639 (PMC7250451; doi:10.1371/journal.pone.0233639)

**Figure S1**. Correlogram of urinary excretion of the 34 signature proteins from the discovery cohort. Pearson correlations of log-transformed protein label-free quantification (LFQ) intensities are shown before the protein header. *P* values are shown above the header.


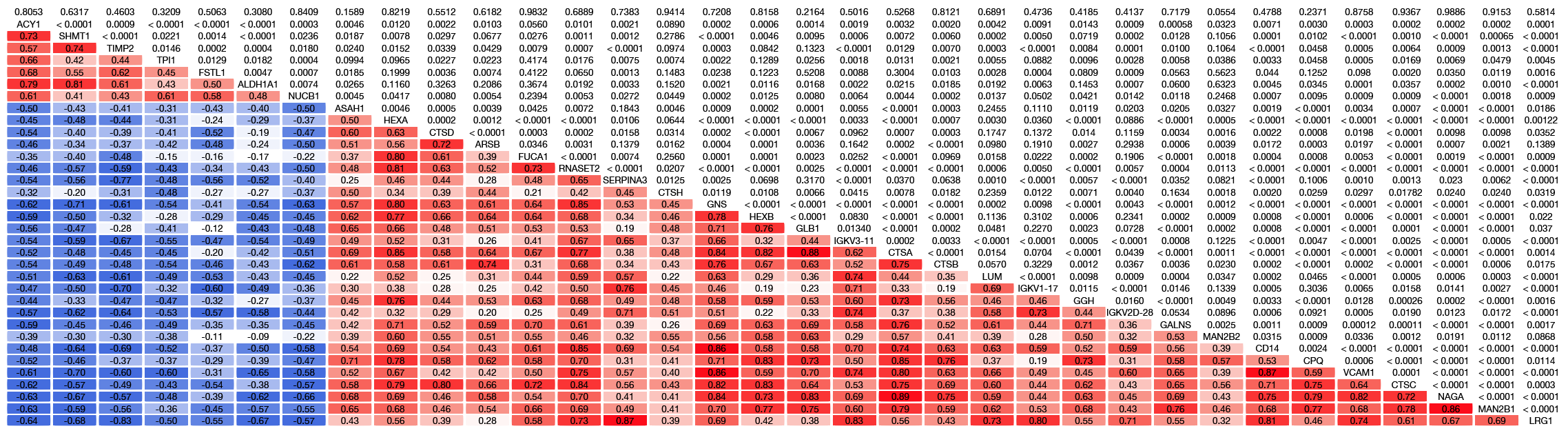

Supplement: S1 Fig — Pearson correlations of log-transformed protein label-free quantification (LFQ) intensities are shown before the protein header. P values are shown above the header. (DOCX) [file pone.0233639.s008.docx]
