# Supplementary material for: Urinary proteomics links keratan sulfate degradation and lysosomal enzymes to early type 1 diabetes
Source: PLoS One. 2020 May 26;15(5):e0233639. doi: 10.1371/journal.pone.0233639 (PMC7250451; doi:10.1371/journal.pone.0233639)

**Figure S3.** Forest plots of fold changes of urinary excretion of the five enzymes associated with keratan sulfate degradation and lumican, a core protein of keratan sulfate proteoglycan. Fold change is calculated as a ratio of the median excretion of youths with diabetes to the that of youths without diabetes. Red points indicate that the protein belong to the urinary signature of the respective study (*Q* < 0.05). Purple points indicate that differential excretion was statistically significant (*P* < 0.05), but not post Benjamini-Hochberg adjustment. Black points failed to reach statistical significance at any level (*P* ≥ 0.05).


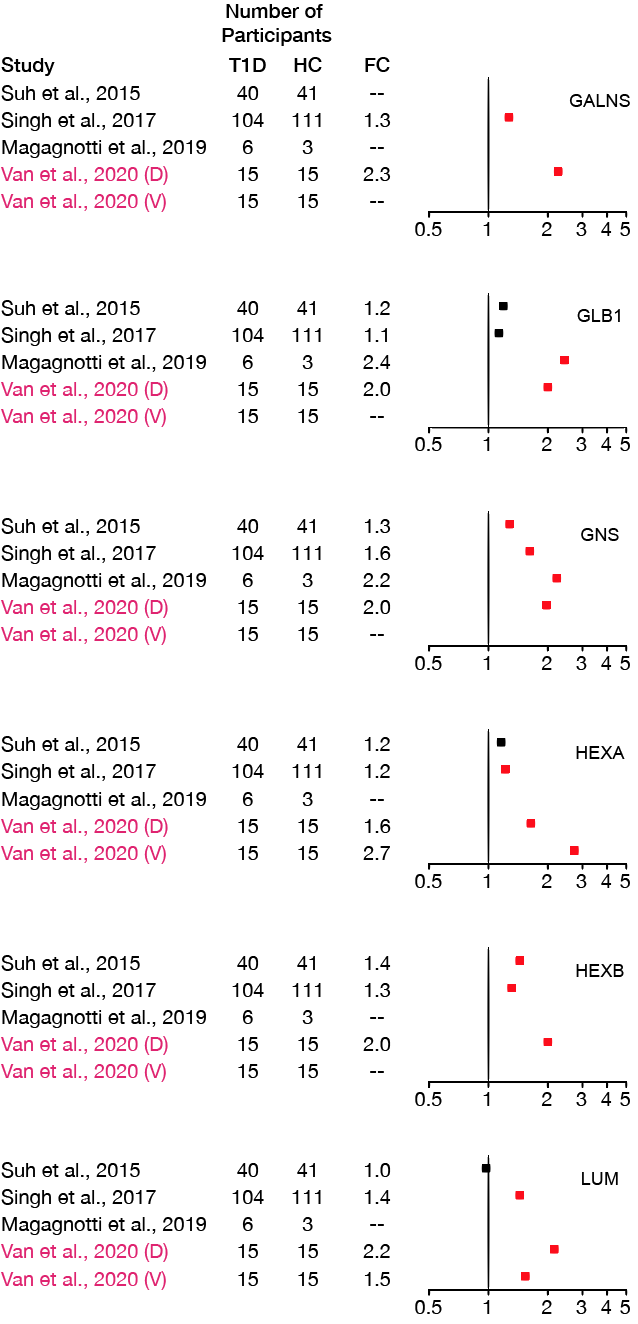

Supplement: S2 Fig — Pearson correlations of log-transformed protein intensities are shown before the protein header. P values are shown above the header. (A) Sub-analysis of the five enzymes associated with keratan sulfate degradation and lumican, a core protein of keratan sulfate. Data is from the discovery cohort. (B) Sub-analysis of the four proteins selected for internal validation and six urinary cytokines/chemokines. Data is from the validation cohort. (C) Sub-analysis of the four proteins selected for internal validation. Data is from the discovery cohort. (DOCX) [file pone.0233639.s009.docx]
